# Safety and Learning Curve of Robotic Rectal Surgery Performed by Young Non-Endoscopic Surgical Skill Qualification System (ESSQS)-Qualified Surgeons: A Single-Center Retrospective Study

**DOI:** 10.7759/cureus.107445

**Published:** 2026-04-21

**Authors:** Hiroshi Saito, Masanori Kotake, Daisuke Fujimori, Daisuke Yamamoto, Masahiro Hada, Takuo Hara, Noriyuki Inaki

**Affiliations:** 1 Department of Surgery, Kouseiren Takaoka Hospital, Takaoka, JPN; 2 Department of Gastrointestinal Surgery/Breast Surgery, Graduate School of Medical Science, Kanazawa University, Kanazawa, JPN; 3 Department of Surgery, Toyama City Hospital, Toyama, JPN

**Keywords:** colorectal, learning curve, rectal cancer, robotic surgery, unqualified surgeon

## Abstract

Background: The number of robotic rectal surgeries being performed in Japan is rapidly increasing. Therefore, the training of young surgeons in robotic rectal surgery has received significant attention. We herein examined the safety and short-term clinical outcomes of robotic rectal surgery performed by young non-Endoscopic Surgical Skill Qualification System (ESSQS)-qualified surgeons.

Methods: Patients who underwent robotic surgery for rectal cancer between November 2019 and October 2023 were included. Robot-assisted rectal surgeries were performed from February 2022 by two young non-ESSQS-qualified surgeons. Young surgeons were defined as those within 12 years after medical graduation. The clinical outcomes of robotic rectal surgery performed by non-ESSQS-qualified surgeons and by qualified surgeons were evaluated. Patients who received preoperative chemoradiotherapy or required complex procedures were excluded.

Results: A total of 269 patients underwent robotic surgery for rectal cancer, and 178 were investigated. We included 35 (19.7%) and 143 (80.3%) patients who underwent robotic surgery by non-ESSQS-qualified surgeons and qualified surgeons, respectively. Median total operative time was longer in the non-qualified group (334 vs. 285 minutes, p<0.001), but postoperative complication rates (6% vs. 10%, p=0.67) and hospital stay (10 vs. 10 days, p=0.85) were similar.

Conclusions: Robotic rectal surgery by young non-ESSQS-qualified surgeons appears feasible with acceptable short-term outcomes, though longer operative times and the need for proctor supervision should be considered.

## Introduction

The number of robotic surgeries for colorectal cancer in Japan has markedly increased since the inclusion of colon cancer in the national health insurance system in April 2022 [1]. Therefore, there is growing interest in education for young surgeons in robotic surgery. Some of the advantages of robotic surgery include 3D visualization, multi-jointed capabilities, and scope stabilization, which may reduce stress on surgeons during surgery. Previous studies demonstrated that the learning curve for robotic surgery in rectal cancer was faster than that for laparoscopic surgery [2-5]. Furthermore, although laparoscopic experience may contribute to the acquisition of basic surgical skills, its impact on the learning curve and clinical outcomes in robotic rectal surgery appears to be limited. This may be attributable to the technical advantages of robotic systems, including enhanced dexterity and improved visualization [6].

In Japan, the Japan Society for Endoscopic Surgery (JSES) has proposed guidelines to prevent severe intraoperative and postoperative complications of robotic surgery [7], and a proctor system for robot-assisted surgery has been introduced for prostatic, gastric, colon, and rectal cancers [8]. Originally, surgeons practicing robotic surgery had to comply with the Endoscopic Surgical Skill Qualification System (ESSQS) as a criterion for safe performance [9]. The ESSQS is a certification program established in Japan to assess and ensure the technical proficiency of surgeons in endoscopic surgery through objective evaluation of surgical performance, and it is also regarded as an indicator of competency as a surgical educator in laparoscopic surgery. However, in March 2020, these guidelines were revised such that non-ESSQS-qualified surgeons who assist in ≥20 robotic surgeries can perform robotic surgery under the guidance of a certified proctor [10]. Since then, young non-ESSQS-qualified surgeons are being increasingly given the opportunity to perform surgery using the robotic system. However, limited information is currently available on the learning curve or postoperative outcomes of robotic surgery performed by non-ESSQS-qualified surgeons. Therefore, we herein examined whether robotic rectal surgery performed by young non-ESSQS-qualified surgeons provides comparable short-term outcomes and safety to that performed by qualified surgeons.

## Materials and methods

Introduction of robotic rectal surgery in our department

Robotic surgery using the da Vinci® Xi surgical system (Intuitive Surgical, Inc., Sunnyvale, CA, USA) for rectal cancer was introduced in our institution in November 2019. Since then, robotic surgery has been indicated for all cases of rectal cancer. A second surgeon began performing robotic surgery from the 49th case, and young non-ESSQS-qualified surgeons from the 147th case (from February 2022). Young surgeons were defined as those within 12 years after medical graduation. Before the introduction of robotic rectal surgery, one of the young surgeons had no experience with laparoscopic rectal surgery, and the other had experience with 10 cases. We introduced robotic surgery for colon cancer in April 2022. Robotic surgery is currently being performed under the supervision of one proctor. All procedures were conducted under the supervision of a certified proctor. The proctor was present throughout the procedures and provided intraoperative guidance using an annotation system and verbal instructions when necessary. The level and nature of proctor involvement were generally consistent between the two groups; however, more frequent guidance was provided to less experienced surgeons as needed to ensure procedural safety. The proctor intervened directly when required. The baseline surgical experience of each surgeon is summarized in Table 1. All surgeons, including ESSQS-qualified surgeons, had no prior experience as console surgeons before the introduction of robotic surgery at our institution. However, ESSQS-qualified surgeons had extensive experience in laparoscopic colorectal surgery. All non-ESSQS-qualified surgeons had participated in robotic procedures as patient-side assistants prior to performing robotic surgery.

**Table 1:** Surgeon characteristics and prior surgical experience. None of the surgeons had prior experience as a console surgeon in robotic rectal surgery before the start of this study. ESSQS: Endoscopic Surgical Skill Qualification System

Surgeon	ESSQS status	Years after graduation	Robotic experience (console, prior to first case)	Robotic experience (assistant, prior to first case)	Laparoscopic colorectal cases
A	Qualified	22	0	0	500
B	Qualified	19	0	48	133
C	Qualified	19	0	7	150
D	Non-qualified	7	0	61	0
E	Non-qualified	9	0	22	10

Study design

The present study was approved by the Institutional Ethical Review Board. The medical records of patients who underwent robotic surgery for rectal cancer between November 2019 and October 2023 were retrospectively reviewed. The first 20 cases following the introduction of robotic surgery were excluded to minimize the impact of the initial learning phase. Exclusion criteria included cases that received preoperative chemoradiotherapy or underwent splenic flexure mobilization, multiple organ resection, lateral lymph node dissection, or intersphincteric resection. The da Vinci® Xi surgical system was installed in our institution. Patient characteristics, such as age, sex, and body mass index (BMI), American Society of Anesthesiologists (ASA) scores, and clinical outcomes, including the total operative time, surgeon console time, blood loss, conversion rate to open surgery, postoperative complications, and postoperative hospital stay, were recorded. The console time included the time from robot docking to specimen extraction. Intraoperative blood loss was recorded by operating room staff, including scrub nurses and anesthesiologists, based on standard intraoperative measurement methods.

There were two young non-EESQS-qualified surgeons described above and three qualified surgeons who have been practicing for more than 15 years after graduation. In our institution, robotic surgery was performed under the supervision of a certified proctor. Intraoperative guidance was consistently provided using an annotation system throughout the study period. The proctor provided real-time instructions as needed; however, direct intervention in the console operation was limited, as a dual-console system was not available. Young surgeons were permitted to serve as console surgeons after accumulating experience as patient-side assistants, completing at least 30 hours of simulator training, and demonstrating sufficient understanding of the surgical procedure under supervision. Postoperative complications were assessed using the Clavien-Dindo classification [11]. Cases were assigned based on surgeon availability, with no formal randomization. More complex cases, such as those requiring splenic flexure mobilization, multiple organ resection, lateral lymph node dissection, or intersphincteric resection, were generally managed by ESSQS-qualified surgeons, which may have introduced selection bias as a potential confounding factor. Perioperative management followed a standardized institutional clinical pathway.

The present study was approved by the Ethical Review Committee of Koseiren Takaoka Hospital (Approval No. 20221222006). Written consent has been obtained from all patients.

The cumulative sum (CUSUM) method

The learning curve was analyzed using the CUSUM method [6,12-14]. CUSUM is the running total of differences between individual data points and the mean of all data points. We applied CUSUM to the console time for each surgeon. Patients were chronologically arranged from the earliest to the latest data on surgery. CUSUM for the first case was the difference between the console time for the first case and the mean console time for all cases. CUSUM for the second case was CUSUM for the previous case, added to the difference between the console time for the second case and the mean console time for all cases. This same procedure was repeated for each patient, except for the last case, which was calculated as zero. The overall mean console time across all surgeons was used as the reference value for the CUSUM analysis. CUSUM was applied descriptively to assess trends in operative performance over time, and no predefined threshold was used to define the completion of the learning curve.

Statistical analysis

Statistical analyses were conducted using EZR (Saitama Medical Center, Jichi Medical University, Saitama, Japan) [15]. Continuous variables were presented as a median (range) and discrete variables as percentages. A p-value <0.05 was considered to be significant.

## Results

Patient characteristics

**Figure 1:**
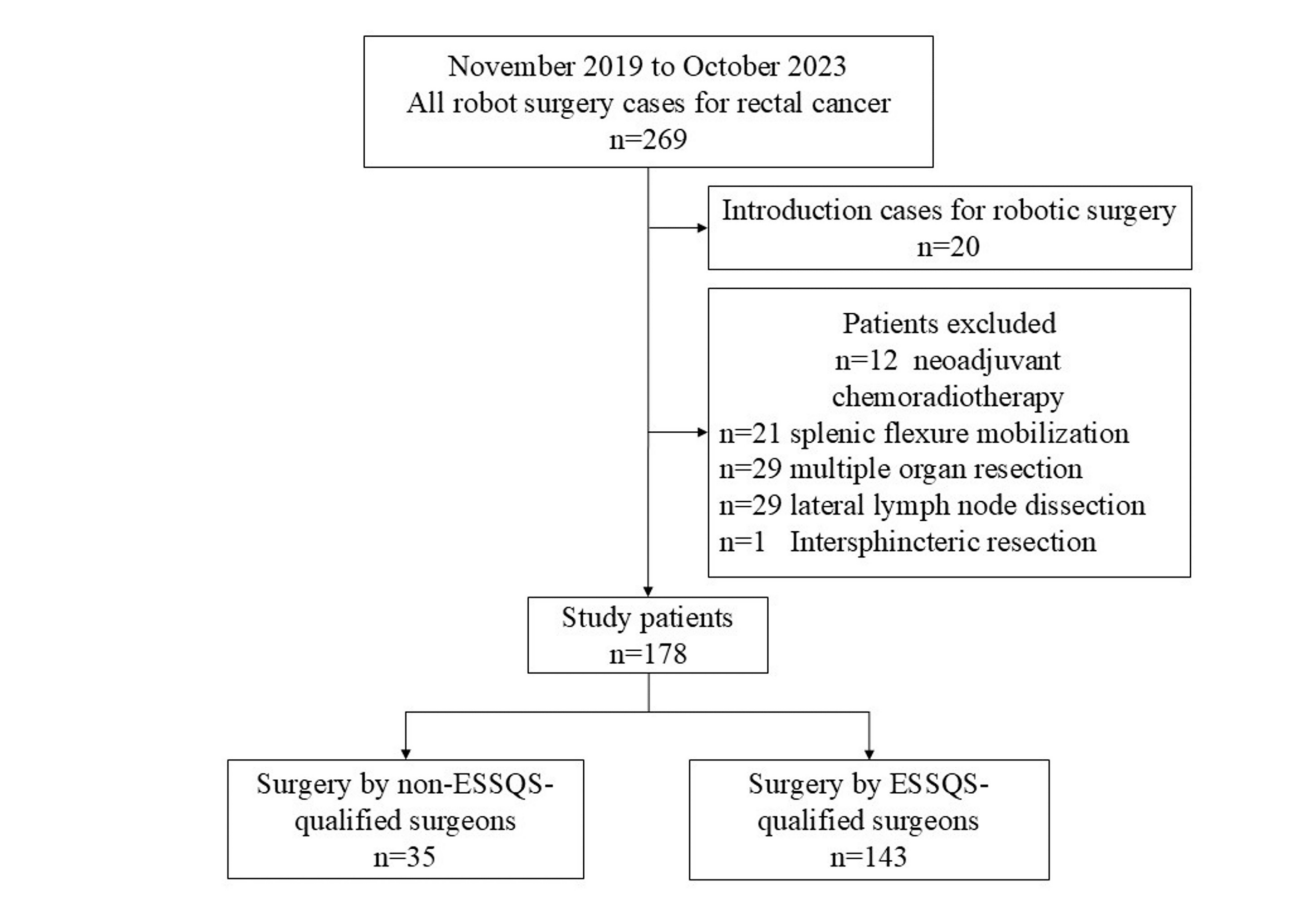
Flow diagram. ESSQS: Endoscopic Surgical Skill Qualification System

Between November 2019 and October 2023, 269 patients underwent robotic surgery for rectal cancer in our institution. Based on the selection criteria, 178 patients were included in the analysis. Of these, 35 cases were operated on by young non-ESSQS-qualified surgeons and 143 by qualified surgeons (Figure 1). No significant differences were observed in age, sex, BMI, ASA scores, or operative method between the two groups (Table 2). The distribution of operative procedures for each surgeon is shown in Table 3.

**Table 2:** Patient characteristics. BMI: body mass index; ASA: American Society of Anesthesiologists

Characteristics	Non-qualified (n = 35)	Qualified (n = 143)	Statistic	P-value
Median age	73 (47-93)	74 (37-94)	W = 2724	0.42
Male, n (%)	20 (57.1)	84 (58.7)	χ^2^ = 0.00	1
BMI, median (range)	22.5 (12.4-34)	22.2 (14.5-30.7)	W = 2586	0.76
ASA score			χ^2^ = 1.96	0.58
1	0	1		
2	29	127		
3	6	14		
4	0	1		
Operative method			χ^2^ = 2.74	0.43
Anterior resection	18	65		
Low anterior resection	10	60		
Abdominoperineal resection	2	4		
Hartmann’s operation	5	14		

**Table 3:** Operative procedures performed by each surgeon.

Surgeon	Anterior resection	Low anterior resection	Abdominoperineal resection	Hartmann’s procedure	Total
A	27	47	3	11	88
B	18	5	1	1	25
C	20	8	0	2	30
D	10	5	2	1	18
E	8	5	0	4	17

Surgical outcomes

Table 4 shows surgical outcomes. The median total operative time was 334 minutes (range, 234-568 minutes) in the young non-ESSQS-qualified surgeons’ group and 285 minutes (range, 140-525 minutes) in the qualified surgeons’ group (p < 0.001). The median console time was 213 minutes (range, 102-436 minutes) in the non-qualified surgeons’ group and 180 minutes (range, 72-402 minutes) in the qualified surgeons’ group (p < 0.001). Median blood loss did not significantly differ between the groups (3 vs. 3 mL, respectively; p = 0.17). The conversion rate to open surgery was 0% in both groups. Furthermore, no significant differences were observed in postoperative complications (6% vs. 10%, respectively; p = 0.67) or the postoperative hospital stay (10 vs. 10 days, respectively; p = 0.85) between the groups.

**Table 4:** Surgical outcomes. Continuous variables are presented as median (range), and categorical variables as n (%). CD: Clavien-Dindo classification

	Non-qualified (n = 35)	Qualified (n = 143)	Statistic	P-value
Median total operative time (min)	334 (234-568)	285 (140-525)	W = 1603	<0.001
Median console time (min)	213 (102-436)	180 (72-402)	W = 1511	<0.001
Median blood loss (mL)	3 (1-50)	3 (1-50)	W = 2143	0.17
Conversion rate	0	0	-	-
Postoperative complications				
≥CD grade 2	2 (6%)	14 (10%)	χ^2 ^= 0.18	0.67
≥CD grade 3	0 (0%)	3 (2%)	χ^2^ = 0.02	0.90
Postoperative hospital stay (days)	10 (7-35)	10 (6-57)	W = 2452	0.85

Learning curve

**Figure 2:**
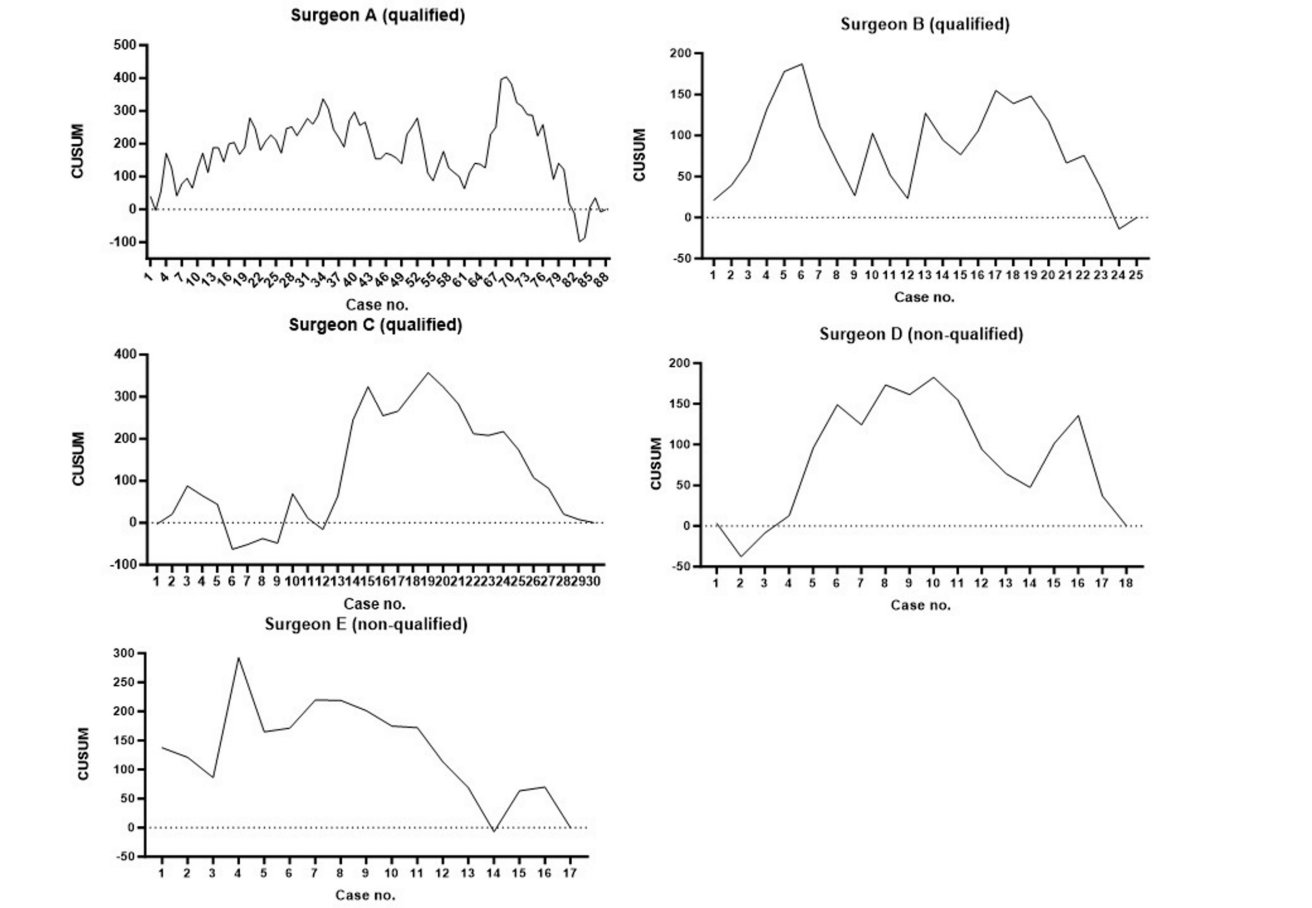
A CUSUM analysis of console times of individual surgeons. CUSUM: cumulative sum

A CUSUM analysis was performed on patients who underwent robotic rectum surgery performed by surgeons A, B, and C (qualified surgeons) and D and E (young non-ESSQS-qualified surgeons) (Figure 2). Console times by surgeon A were similar until the 34th case and then decreased. Console times by surgeon B decreased after the sixth case, gradually increased to the 17th case, and then decreased again. Console times by surgeon C increased to a peak by the 19th case and then gradually decreased. Console times by surgeon D increased to a peak by the 10th case and then gradually decreased. Console times by surgeon E were similar until the seventh case, and then gradually decreased.

## Discussion

In Japan, robotic rectal surgery was included in the national health insurance system from 2018, and colon cancer surgery from 2022. The introduction of robotic surgery is advancing at each institution, and the number of cases is markedly increasing [1]. The qualifications of a console surgeon for robotic surgery are stipulated by the JSES. The initial requirement to introduce robotic surgery was to become a specialist in the Japan Society of Gastroenterological Surgery and an EESQS-qualified surgeon. However, these requirements have gradually been relaxed, and robotic surgery may now be performed regardless of this qualification as long as it is conducted under the guidance of a proctor. Based on the short-term outcomes of robotic rectal surgery, robotic surgery is performed more safely than laparoscopic surgery. Yamaguchi et al. suggested that it was superior in terms of blood loss, the length of hospital stay, and postoperative complications [16,17].

The learning curve for robotic rectal cancer surgery was found to peak after 21-35 cases, which was significantly lower than the 50-90 cases reported for the learning curve for the laparoscopic approach [2,13,18-20]. With the further expansion of robotic surgery in the future, opportunities for young non-ESSQS-qualified surgeons to perform surgeries will increase. However, there is currently no information on the short-term outcomes of robotic surgery performed by young surgeons with limited experience of laparoscopic surgery. Therefore, we herein investigated the short-term outcomes of robotic surgery performed by young non-ESSQS-qualified surgeons and also examined its validity and safety.

The results obtained showed that operative and console times were longer by young non-ESSQS-qualified surgeons than by qualified surgeons, whereas conversion rates, postoperative complication rates, and the postoperative hospital stay were similar. Regarding the learning curve, even young non-ESSQS-qualified surgeons with limited experience of laparoscopic surgery completed the learning curve for robotic rectal surgery relatively early. As part of the education process for robotic surgery in our institution, young surgeons initially become proficient in robotic surgery as a patient-side assistant before performing surgeries as the console surgeon. Training as an assistant and repeatedly observing and learning from attending qualified surgeons has been suggested to allow surgeons with limited experience of laparoscopic surgery to safely perform robotic surgery under the guidance of a proctor. Noh et al. [6] noted that the number of experiences of laparoscopic surgery had a limited impact on the learning curve for robotic rectal surgery, and further investigations are expected in the future. It is also necessary to conduct studies on long-term outcomes. A previous study noted the safety of robotic distal gastrectomy performed by non-ESSQS-qualified surgeons in a single-center, retrospective analysis [9]. It will be necessary to conduct similar investigations on many operative methods within the field of gastrointestinal surgery. The relatively low intraoperative blood loss observed in both groups may reflect the precise dissection enabled by robotic surgery and the standardized intraoperative measurement by operating room staff. However, this finding should be interpreted with caution when compared with other studies.

This study has several limitations. First, case allocation was not randomized, and although no intentional selection bias was applied, unmeasured confounding factors may have influenced the results. In particular, less complex cases may have been preferentially assigned to non-ESSQS-qualified surgeons, potentially affecting the comparability between groups. Second, the sample size was small, particularly in the young non-ESSQS-qualified surgeons’ group, which may have limited the statistical power to detect significant differences in clinical outcomes. In addition, the imbalance in sample size between the two groups may have affected the comparability of the results. Third, the potential impact of temporal bias cannot be excluded, as robotic surgery was introduced sequentially, and surgical experience, perioperative management, and institutional proficiency may have improved over time. Surgical performance may also improve over time regardless of surgeon qualification. This factor was not adjusted for in the present study and should be considered when interpreting the findings. In addition, differences in operative procedures between the groups may have influenced surgical difficulty and postoperative outcomes. Because no adjustment for baseline characteristics was performed, this factor may represent a potential confounder. Fourth, all procedures were performed under the supervision of an experienced proctor; therefore, our results may reflect not only the performance of the individual surgeons but also the effect of structured supervision and guidance. In addition, a dual-console system was not available at our institution, which may have influenced the learning process. Furthermore, potential differences in the extent or frequency of proctor involvement between groups may have also influenced the observed outcomes. Fifth, we did not evaluate long-term oncological or functional outcomes, such as recurrence rates or postoperative urinary and sexual function, which are important endpoints in rectal cancer surgery. Finally, this was a retrospective single-center analysis, which may limit the generalizability of our findings. Therefore, further large-scale, prospective, multicenter studies are required to validate the safety and feasibility of robotic rectal surgery performed by non-ESSQS-qualified surgeons.

## Conclusions

Robotic rectal surgery appears feasible in young non-ESSQS-qualified surgeons when performed under structured supervision. Although operative and console times were longer than those of qualified surgeons, no significant differences were observed in short-term perioperative outcomes. These findings suggest that outcomes may be influenced by both surgeon experience and institutional supervision, and should not be interpreted as equivalent in all clinical settings, particularly for more complex cases. Furthermore, the learning curve analysis was descriptive using CUSUM without predefined thresholds; therefore, conclusions regarding its completion should be interpreted cautiously. Further large-scale prospective studies are required to validate these findings and to assess long-term outcomes.
